# Modes of Action of 1,8-Cineol in Infections and Inflammation

**DOI:** 10.3390/metabo13060751

**Published:** 2023-06-13

**Authors:** Ralph Pries, Stephanie Jeschke, Anke Leichtle, Karl-Ludwig Bruchhage

**Affiliations:** Department of Otorhinolaryngology, University of Luebeck, 23538 Luebeck, Germany; stephanie.jeschke@uksh.de (S.J.); anke.leichtle@uksh.de (A.L.); karl-ludwig.bruchhage@uksh.de (K.-L.B.)

**Keywords:** 1,8-Cineol, pharmacokinetics, anti-microbial, inflammation, immune regulation

## Abstract

The monoterpene 1,8-Cineol is a natural plant-based therapeutic agent that is commonly applied to treat different inflammatory diseases due to its mucolytic, anti-microbial and anti-inflammatory properties. It has become increasingly clear in the recent years that 1,8-Cineol spreads almost everywhere in the human body after its oral administration, from the gut to the blood to the brain. Its anti-microbial potential and even its anti-viral effects have been observed to include numerous bacteria and fungi species. Many recent studies help to better understand the cellular and molecular immunological consequences of 1,8-Cineol treatment in inflammatory diseases and further provide information concerning the mechanistic modes of action in the regulation of distinct inflammatory biosynthetic pathways. This review aims to present a holistic and understandable overview of the different aspects of 1,8-Cineol in infections and inflammation.

## 1. Origin, Pharmacokinetics and Systemic Distribution

**Figure 1:**
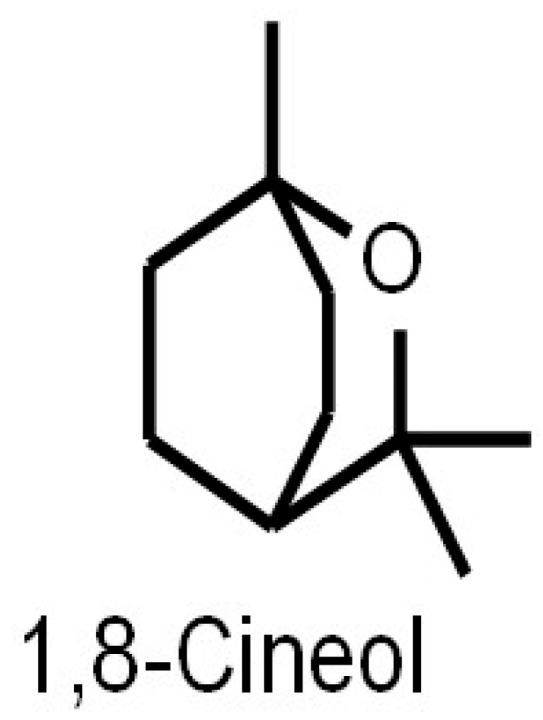
Chemical structure of 1,8-Cineol (C_10_H_18_O). 1,8-Cineol is a liquid, colorless lipophilic with a camphor-like aroma and a density of 0.93 g/cm^3^ (at 20 °C).

The eucalyptus tree (*Eucalyptus* spec.) is the major natural source of the monoterpene 1,8-Cineol (1,3,3-trimethyl-2-oxabicyclo[2.2.2]octane), where it is of importance as a leaf toxin that defends against predation by herbivores such as the brushtail possum (*Trichosurus vulpecula*) [1]. Eucalyptus oil has already long been used by Australian aboriginal natives to treat injuries and inflammation [2]. However, other plants such as oregano (*Origanum* spec.), thyme (*Thymus* spec.), guava (*Psidium pohlianum*) or sage (*Salvia* spec.) contain this secondary plant metabolite as well [3,4,5]. Further investigations revealed different compositions and associated anti-microbial activities of 1,8-Cineol containing essential oils extracted from different *Salvia* species [6]. Eucalyptus oil used for medicinal applications should contain at least 70% of 1,8-Cineol, according to the European Pharmacopoeia [7]. Moreover, the production of 1,8-Cineol by cyanobacteria has recently been published. Sakamaki and colleagues overexpressed the gene encoding the 1,8-Cineol synthase in the cyanobacterium *Synechococcus elongates*, which resulted in an efficient 1,8-Cineol production via photosynthesis and without supplementing any carbon source [8]. Besides its isolation from various natural sources, 1,8-Cineol can also be synthesized in vitro by isomerization of α–terpineol [9]. 1,8-Cineol was first described by Cloez in 1870 [10] and is a liquid and colorless lipophilic with a camphor-like aroma and a density of 0.93 g/cm^3^ (at 20 °C). The molecular formula of Cineol is C_10_H_18_O (Figure 1). 1,8-Cineol has a molecular weight of 154.25 g/mol, a melting point of −1.3 °C and a boiling point of 177 °C [11]. So far, there is only little information in regard to side effects or the cytotoxicity of 1,8-Cineol. 

Evaluation of 1,8-Cineol toxicity revealed values of 2.48 grams per kg in a rat model [12]. Due to potential allergic reactions, it is recommended to avoid applying 1,8-Cineol-rich essential oils to the face or eyes. Because of the low viscosity of 1,8-Cineol, it may directly enter the lungs if swallowed and therefore it it recommended to avoid application to infants and children under the age of 5 [12,13]. Furthermore, gastric distress such as nausea or diarrhea was described as potential side effect in a few patients in a bronchial asthma study, whereas overall compliance was considered good in all patients [14]. Natural plant-based 1,8-Cineol has multiple uses such as that of a food additive, flavoring agent, cosmetic agent and pharmaceutical agent. It is commonly applied to treat various chronic and acute airway diseases [15]. Interestingly, besides its long-established utility in treating sinusitis, bronchitis and chronic obstructive pulmonary disease (COPD) [16], it has also revealed to possess health-protective effects against ethanol-induced gastric mucosal damage [17].

The bioactivity of 1,8-Cineol is known to be rather limited due to its low aqueous solubility and stability; therefore, a regular ongoing administration is required [18]. The metabolization of 1,8-Cineol is maintained by the cytochrome P450 system in the mammalian liver [19,20] and the associated metabolites (2-hydroxy-, 3-hydroxy-, 7-hydroxy- and 9-hydroxy-1,8-Cineol) can be detected accordingly in plasma and urine samples following the uptake of 1,8-Cineol [21]. In vitro investigations using human liver microsomes and recombinant cytochrome P450 enzymes corroborated a clear correlation between the incubation time and enzyme content and the resulting metabolization and concentrations of 1,8-Cineol metabolites [19].

Earlier pharmacokinetic studies revealed that 1,8-Cineol is efficiently absorbed from breathing air upon inhalation and is detectable in the blood plasma after approximately 18 min [22]. The quick and efficient absorption of 1,8-Cineol was also shown in a rat pharmacokinetic study where the serum concentration time profiles of 1,8-Cineol indicated that the absorption characteristics after an oral administration are similar compared to an intravenous administration [23].

Hence, the systemic distribution of 1,8-Cineol in the human body and the consequences for the associated direct and indirect therapeutic effects are becoming increasingly clear.

**Figure 2:**
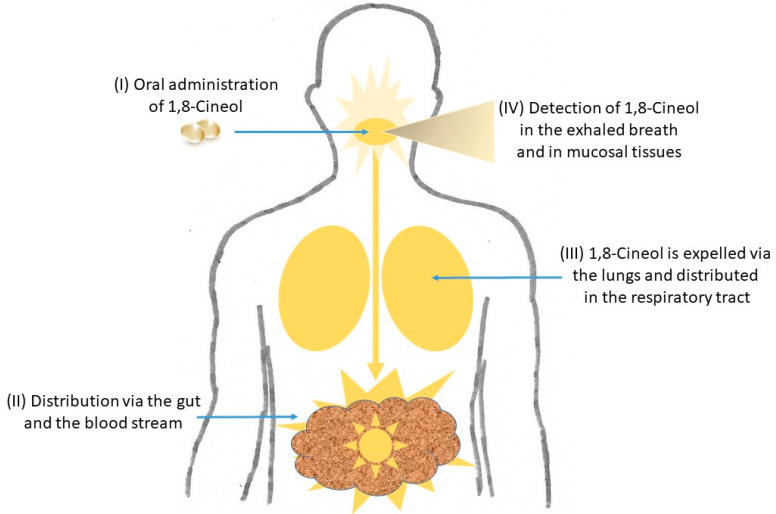
Distribution of 1,8-Cineol in the human body. After its oral administration as enteric coated capsules and the passage through the stomach, the systemic distribution of 1,8-Cineol occurs via the gut and the blood stream to the respiratory tract. It can finally be detected in the exhaled breath gas as well as in mucosal tissues. See text for details.

Different 1,8-Cineol containing medications are known to be applied orally as enteric coated capsules and their curative effects evolve after their passage through the stomach within the small intestine. It has recently been shown that 1,8-Cineol was detectable in nasal tissue samples after its oral administration for 14 days, which indicates the systemic distribution of 1,8-Cineol via the gut and the blood stream [24] (Figure 2).

Subsequently, 1,8-Cineol is expelled from the lungs and can unfold its anti-inflammatory effects in the respiratory tract and the mucosal tissues, which has been monitored by real-time breath gas analysis using online proton-transfer-reaction mass spectrometry (PTR-MS) [25] (Figure 2). In a recent publication, the oral application of 1,8-Cineol containing nanoemulsions was shown as a promising systemic therapeutic approach for patients with atherosclerosis, because this administration form of 1,8-Cineol resulted in an increased stability and prolonged its retention time in the gastrointestinal tract [26].

Reduced inflammatory parameters have also been shown in airways of ovalbumin-triggered guinea pigs upon 1,8-Cineol treatment by inhalation, which impaired the development of airway hyper-responsiveness [27].

Therefore, 1,8-Cineol is routinely applied as an alternative option to treat chronic rhinosinusitis with nasal polyps (CRSwNP), whereas the administration of corticosteroids or antibiotics and surgery are still the most established therapies of CRSwNP [28].

CRSwNP is a common disease worldwide, affecting about 10% of the European population, and is frequently associated with asthma and allergic rhinitis [29]. Various factors are known to be associated with CRSwNP, such as air pollution, individual immune barrier dysfunctions or alterations in the eicosanoid pathway, whereas microbial pathogens such as *Staphylococcus aureus* are suspected to play a key role in the development of CRSwNP and are supposed to be affected by 1,8-Cineol [30,31,32].

## 2. Anti-Microbial and Anti-Viral Potential

An anti-microbial activity of 1,8-Cineol has been described in different inflammatory diseases [33,34,35,36]. An important aspect of natural anti-microbial compounds is the lower risk of antibiotic resistance development. A strong anti-microbial effect of 1,8-Cineol in co-administration with chlorhexidine gluconate was observed with regard to methicillin-resistant *S. aureus* strains and also for *E. coli*, *K. pneumoniae*, *E. faecalis* and *C. albicans* strains with a weaker effect [37]. Essential oils extracted by hydrodistillation from the leaf parts of *E. globulus* revealed significant anti-bacterial properties and caused highly significant decreased microbial counts of *S. aureus* in agar diffusion assays [38]. However, bacteria-induced inflammatory skin disorders also revealed recovery effects upon treatment with 1,8-Cineol-containing compounds. A suppression of *Propionibacterium acnes*-induced skin inflammation was shown in response to bay tree (*Laurus nobilis*) extracts and its major constituent, eucalyptol [39]. Similar results were obtained by a combination of essential oils from spice plants *Amomum verum* Blackw. and *Zanthoxylum limonella* against different bacterial species (*Staphylococcus aureus*, *Staphylococcus epidermidis*, *Escherichia coli* and *Pseudomonas aeruginosa*) with 1,8-Cineol and limonene as the essential anti-bacterial components [40].

Recently, essential oils from *Eucalyptus globulus* with 1,8-Cineol as the main metabolite (65.83%) showed anti-microbial activity against *Streptococcus mutans* even in biofilm cultures, emulating dental plaque conditions [41]. Biofilm formation is a major limiting factor concerning the efficacy of anti-microbial treatment due to low penetrability. 1,8-Cineol has been shown to penetrate *E. coli* biofilm, which could be enhanced under osmotic stress [42]. These promising results are of great importance for inflammation treatment and also livestock farming, where it is necessary to reduce the level of antibiotics and to prevent the development of multidrug resistance. In this context, anti-bacterial activity of 1,8-Cineol was demonstrated against various strains of *E. coli* (including Avian Pathogenic *E. coli*; APEC) isolated from broiler chicken [43]. However, further investigations concerning the anti-microbial activity revealed that the fruit oil of *E. globulus* was hardly active against multidrug-resistant (MDR) Gram-negative bacteria [44,45]. Acknowledged limitations of the clinical application of 1,8-Cineol are its instability, the short half-life and its vitality. In order to improve the bioavailability of natural plant-derived compounds, the preparation of invasomes (spherical vesicles of phospholipid bilayers) encapsulating thymol, menthol, camphor and 1,8-Cineol has recently been suggested as a promising approach to efficiently treat bacterial infections [46].

Acknowledged limitations of the clinical application of 1,8-Cineol are its instability, the short half-life and its vitality. Moreover, it has been shown in a rat skin model that a high bioavailability of 1,8-Cineol could be obtained using a microemulsion gel for its transdermal delivery [47].

Of note, investigations on pathogenic *S. aureus*, the most abundant bacterial species in chronic rhinosinusitis, revealed a growth inhibitory effect of 1,8-Cineol, but also downregulation proteins involved specifically in biofilm formation [36]. This is a very interesting aspect, since the QS (quorum sensing) pathway is known to be required for proper formation and functioning of bacterial biofilms. QS is the regulatory communication system via chemical signals within bacterial and fungal populations and is involved in invasion, defense and distribution [48,49]. In this context, 1,8-Cineol has been shown to modulate QS-related bacterial receptors [50] as well as the cellular characteristics of the bacterial shape and size [51].

Moreover, it has recently been shown that 1,8-Cineol contributes to a synergistic anti-microbial and also anti-fungal activity within a novel substance based on *Melaleuca alternifolia* leaf oil. The major biologically active compounds were terpinen-4-ol (20.88%), 1,8-Cineol (22.28%), (-)-α-bisabolol (25.73%) and o-cymene (8.16%). This substance revealed inhibitory effects against the yeast *Malassezia furfur*, which is a main pathogen involved in the pathogenesis and manifestation of the dermatological disease *Seborrheic dermatitis* [52].

Further, 1,8-Cineol was suggested as an alternative to conventional fungicides against different *Penicillium* species. Data revealed that fungal citrus pathogens developed a tolerance to glyphosate and conventional fungicides but not to 1,8-Cineol [53].

In addition to its anti-bacterial and anti-fungal potential, there is also evidence that 1,8-Cineol appears to be able to augment protection against viral infections.

It has been observed that an intra-nasal co-administration of 1,8-Cineol together with influenza vaccine provides a cross-protection against influenza virus infections. In the underlying study, mice were intra-nasally treated three times with two doses of 1,8-Cineol (6.25 and 12.5 mg/kg) over two weeks (day 0, 7, 14) and infected with influenza virus 7 days later. Mice that had received a co-administration of 1,8-Cineol showed longer survival, milder signs of inflammation and increased levels of influenza-specific serum immunoglobulin (Ig) G2a and respiratory tract intraepithelial lymphocytes (IELs) in the upper respiratory tract [54]. Moreover, 1,8-Cineol revealed the potential to protect against influenza virus-induced pneumonia and efficiently decreased the levels of inflammatory cytokines such as of IL-4, IL-5, IL-10 in nasal lavage fluids and the levels of IL-1β, IL-6, TNF-α and IFN-γ in lung tissues in a corresponding mouse model [55]. In this context, it has been shown that 1,8-Cineol potentiates the activity of the anti-viral transcription factor interferon regulatory factor 3 (IRF3) in different human cell lines and in cultivated human nasal mucosa [56].

## 3. Cellular Consequences in Response to 1,8-Cineol

1,8-Cineol is increasingly perceived as a non-prescription mucolytic medication in virus- or bacteria-associated inflammatory diseases such as bronchitis or chronic obstructive pulmonary disease (COPD) [16]. Besides its anti-microbial effects, the influence of 1,8-Cineol on various different human cell types and the regulation of associated molecular consequences have been observed. Respiratory inflammatory diseases such as chronic rhinosinusitis or bronchial asthma are known to be accompanied by increased mucus secretion levels from epithelial cells in the respiratory tract. It has been shown in in vitro cultures of human nasal turbinate slices with lipopolysaccharides (LPS; mimicking bacterial infection) that the numbers of mucin harboring goblet cells significantly decreased in response to 1,8-Cineol treatment. These data were corroborated by real-time PCR analysis that further showed significantly reduced expression levels of the mucin genes MUC2 and MUC19 in close association with a significantly attenuated activity of transcription factor NF-κB [57]. Similarly, it was shown that 1,8-Cineol treatment against influenza virus infection in mice reduced the expression levels of transcriptional activator nuclear factor (NF)-kB p65 and expression of intercellular adhesion molecule (ICAM)-1 and vascular cell adhesion molecule (VCAM)-1 in lung tissues [55]. Investigations in colon cancer cells on the potential genotoxicity of 1,8-Cineol revealed a concentration-dependent increase in oxidative DNA damage, whereas it did not affect the cell viability due to DNA repair mechanisms [58].

In addition, 1,8-Cineol containing eucalyptus oil was shown to decrease allergic reactions by suppressing the degranulation of mast cells as well as suppressing the expression of lipid mediators and prostaglandin D2 [59]. In this context, the airway hyper-responsiveness of bronchial epithelial cells in responses to house dust mite provocation was significantly reduced by intra-nasal 1,8-Cineol treatment and was associated with decreased expression levels of different inflammatory cytokines such as interleukin (IL)-4, IL-6 and granulocyte macrophage colony stimulating factor (GM-CSF) in bronchial epithelial cells [60]. Comparable influences of 1,8-Cineol could be detected in immune cells, where a significantly reduced expression of pro-inflammatory mediators such as TNF-α, IL-1β and IL-6 from monocytes as well as the IL-4 and IL-5 production from lymphocytes [61] were observed. Even in the inflammatory skin disorder acne, 1,8-Cineol-containing leaf extracts significantly suppressed the expression of pro-inflammatory cytokines IL-1β and IL-6 [39]. Moreover, treatment with 1,8-Cineol in combination with ellagic acid has been shown to downregulate different cytokines such as transforming growth factor beta-1 (TGF-β1), Fascin-1 (FSCN1), vascular endothelial growth factor (VEGF) and matrix metalloproteinase-9 (MMP-9) in patients with hepatocellular carcinoma (HCC) [62].

Thus, it is becoming increasingly clear that a central mode of action of 1,8-Cineol is the inhibition of pro-inflammatory cytokine expression and its immunological consequences.

It was recently shown in CRSwNP patients that not only cytokine expression patterns, but also abundances of circulating immune cell subsets are affected by 1,8-Cineol. Data revealed a decrease in classical monocytes accompanied by a significant increase in intermediate CD16^+^ monocytes in the peripheral blood of CRSwNP patients. Chronic rhinosinusitis patients with a severe redistribution of these monocyte subsets revealed a significant restoration in response to two-week 1,8-Cineol treatment [63]. Nitric oxide (NO) is also known to possess airway-modulating functions in patients with chronic rhinosinusitis and an increased phosphorylation in eNOS was shown in nasal polyps from CRSwNP patients, which was significantly inhibited in response to 1,8-Cineol treatment [64]. Further, it has been reported that inhalation of 1,8-Cineol leads to reduced abundances of eosinophils and reduced levels of cytokines IL-4, IL-13 and IL17A in broncho–alveolar lavage fluid in a murine asthma model [60]. The murine lung alveolar macrophages infection model was meant to mimic mycobacterial infections in alveolar macrophages, and eucalyptus oil and its constituent, 1,8-Cineol, proved to significantly enhance phagocytosis activity and mycobacterial clearance [65].

Besides its influence on the proportions and functions of different immune cells, 1,8-Cineol has been recently suggested as an inhibitor of platelet activation, which was non-toxic to platelets up to a concentration of 50 µM in a mouse model. The inhibition of integrin αIIbβ3 signaling by 1,8-Cineol was found to be responsible to prevent thrombus formation in this study [66]. Inhibitory effects of 1,8-Cineol on cancer cells are also evident [67,68]. For instance, 1,8-Cineol was shown to suppress the activation of the MAPK/ERK (extracellular signal-regulated kinase) pathway and phosphorylation of its upstream kinases in skin carcinogenesis and, correspondingly, delayed tumor incidence and reduced tumor numbers have been shown in mice [69]. There are different reports on MAPK pathway inhibition by compounds derived from natural products [70,71].

## 4. Influence on Distinct Biosynthetic Pathways

**Figure 3:**
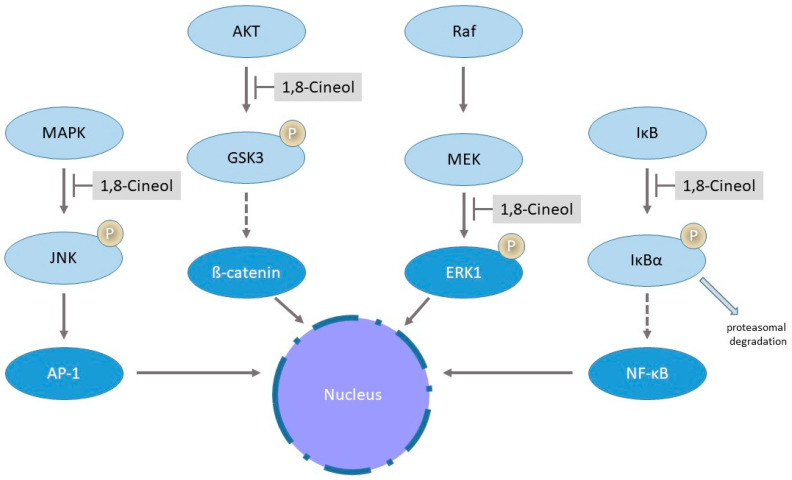
Influence of 1,8-Cineol on distinct biosynthetic pathways. The figure illustrates the schematic phosphorylation (P)-mediated regulatory cascade of the translocation and transcriptional activation of AP-1 (activator protein-1), β-catenin, Egr-1 (early growth response-1) and NFκB (nuclear factor-κB) with regard to the inhibitory impact of 1,8-Cineol. See text for further details.

Mitogen-activated protein kinases (MAPK) are the key regulators of various inflammatory responses and mediate translocation and transcriptional activity of numerous transcriptional activation cascades via phosphorylation. Alterations of MAPKinase signaling has been identified in many different human diseases, whereas the transcriptional nuclear factor-κB (NF-κB) is an important component within these intracellular signaling pathways, leading to the expression of inflammatory mediators [72]. The nuclear translocation of NF-κB depends on the phosphorylation and ubiquitination-dependent proteasomal degradation of IκBα by IκB [73]. The transcriptional activation of numerous target genes via NFκB signaling has been identified as a major pathway involved in various inflammatory diseases such as asthma, rheumatoid arthritis and also in different human cancers [74]. Greiner and colleagues described, for the first time, a significant downregulation of inflammatory processes via decreased activities of transcription factor NFκB and the JNK (c-Jun N-terminal kinase)/AP-1 (activator protein-1) pathway in the human cancer cell lines U373 and HeLa in response to 1,8-Cineol, the active ingredient of the drug Soledum^®^. Data revealed a reduced nuclear translocation of NFκB and correspondingly a decreased transcription of target genes in lipopolysaccharide (LPS)-stimulated cells in the presence of 1,8-Cineol [75]. Inhibition of the nuclear import of NFκB was caused by increased protein levels of IκBα, which can then bind to the NFκB-NLS (nuclear localization signal) and thus inhibit its translocation [75] (Figure 3).

The NFκB-dependent inflammation was also inhibited in patients suffering from *P. acnes*-induced skin disease upon treatment with 1,8-Cineol containing *L. nobilis* extracts [39]. These data were further corroborated by a study using the T24 human bladder epithelial cell line in a TNFα-stimulated inflammation assay, where the extracted oil of *E. globulus* was found to be even more effective in inhibiting NFκB-induced IL-8 secretion than the specific NFκB-inhibitor ACHP (2-amino-6-[2-(cyclopropylmethoxy)-6-hydroxyphenyl]-4-(4-piperidinyl)-3-pyridineca-bonitrile) [76].

In a recent study, the effects of *Lavandula viridis* extracts on ROS (reactive oxygen species) production, inflammatory response and proteasome activity on LPS-stimulated macrophages were investigated. Data indicated an inhibition of nitric oxide production through downregulation of NFκB-related Nos2 transcription and subsequent iNOS protein expression. The anti-inflammatory activity was also indicated by a strong inhibition of LPS-induced pro-inflammatory cytokines IL-1β and IL-6 [77]. Furthermore, it has been shown in an acute lung injury mouse model that 1,8-Cineol suppressed the NFκB-dependent expression of MMP9 (matrix metalloproteinase-9), which belongs to the zinc-metalloproteinases family and is involved in the degradation of the extracellular matrix [78].

Egr-1 (early growth response-1) is another MAPK-pathway-regulated transcription factor that plays an important role in the regulation of many inflammation-associated genes encoding for cytokines, chemokines, cell adhesion molecules and immune receptors [79,80].

1,8-Cineol reduces LPS-induced expression and nuclear translocation of transcription factor Egr-1 via the MEK-extracellular signal-regulated kinase pathway in the human monocyte cell line THP-1 [81] (Figure 3).

Moreover, it has been shown in melanoma that hyperactivated MAPK signaling downregulates the Wnt/β-catenin signal transduction cascade [82]. In this context, it has been shown that 1,8-Cineol acts as an inhibitor of the Wnt/β-catenin pathway in head and neck squamous cell carcinoma (HNSCC). In HNSCC, a dose-dependent decreased cellular progression is associated with a decreased inhibition of glycogen synthase kinase 3 (GSK-3) and reduced levels of WNT11 [68]. GSK-3 is the key enzyme of glycogen metabolism and an important regulator of other inflammatory signaling pathways. It was first described in 1980 and since then, numerous studies have provided evidence to its association with different human diseases such as diabetes mellitus, obesity or chronic rhinosinusitis [83]. 1,8-Cineol also acts as an inhibitor of the Wnt/β-catenin signaling pathway in patients with chronic rhinosinusitis by affecting the AKT-dependent inhibitory phosphorylation of GSK-3, which is the key regulator of the β-catenin activity [35] (Figure 3).

A recent study by Chen and colleagues provides further mechanistic insight concerning the mode of action of 1,8-Cineol in the regulation of Wnt/β-catenin signaling. Data indicated that 1,8-Cineol could inhibit bisphenol A-induced apoptosis and immunosuppression in grass carp hepatocytes by regulating the Wnt/β-catenin signaling pathway through binding to keap1 (Kelch-like ECH-associated protein 1) [84]. Keap1 interacts with Nrf2 (nuclear factor erythroid-2-related factor 2), a key transcriptional regulator of the antioxidant response, which is essential for the amelioration of oxidative stress through decreased levels of ROS and oxidative stress-related indicators [85].

Moreover, 1,8-Cineol has been shown to have neuroprotective activity. The decreased activity of GSK-3 in response to 1,8-Cineol could ameliorate advanced glycation end products, which can be identified by immunohistochemistry in the senile plaques and neurofibrillary tangles of Alzheimer’s disease [86,87]. In this context, it was recently shown that eucalyptol reveals an opening effect on the blood–brain barrier and thus provides a promising addition in the treatment of illnesses of the central nervous system and its brain pharmacokinetics [88].

Besides the regulatory impact of 1,8-Cineol on the translocation and activity of different MAPK-dependent transcriptional activators, it also controls inflammation by suppressing the NOD-like receptor pyrin domain-containing 3 (NLRP3) activation, which is the critical step in the inflammasome formation. NLRP3 is the key regulator of cytokine IL-1β secretion and caspase-1 activity and promotes the pathogenesis of many diseases. It has recently been shown that 1,8-Cineol containing *L. nobilis* leaf extract inhibited NLRP3 activation as well as a decreased activity of the transcription factor NF-κB and p38 of the shared downstream signaling cascade, which underlines the anti-inflammatory potential of 1,8-Cineol [65]. 1,8-Cineole significantly reduced the LPS-induced NLRP3 activation in murine alveolar macrophages, which was associated with a significant increase in phosphorylated ERKs (ERK1/2) and a significant reduction in phosphorylated p38 [65].

Further detailed analysis of the anti-inflammatory mechanism of *L. nobilis* extract revealed that the observed inhibition of NLRP3 inflammasome activation was correspondingly associated with an inhibition of caspase-1 activation and apoptosis-associated speck-like protein containing a CARD (ASC) pyroptosome complex formation [89]. The 1,8-Cineol containing leaves of the plant *Litsea cubeba* were also shown to inhibit the NLRP3 inflammasome activation and to ameliorate the dextran-sulfate-induced colitis [90] and pro-inflammatory cell infiltrations in ankle tissues in mouse models [91].

In order to investigate the impact of 1,8-Cineol on intestinal inflammatory diseases such as bowel disease, Crohn’s disease or ulcerative colitis, dextran sodium sulfate (DSS)-induced colitis was analyzed in mice. Data revealed molecular binding of 1,8-Cineol to the peroxisome proliferator-activated receptor-γ (PPARγ), that plays an important role in the regulation of colonic inflammation [92]. Correspondingly, recent data revealed that 1,8-Cineol mediated inhibition of PPARγ prevents the polarization of M2 macrophages and alleviates bleomycin induced pulmonary fibrosis [93].

Overall, inflammation is known to be key driver of various acute and chronic diseases such as appendicitis, bronchitis or arthritis. Reinforcing anti-inflammatory effects of 1,8-Cineol have recently been shown in combination with the non-steroidal anti-inflammatory drug flurbiprofen, which resulted in an increased downregulation pro-inflammatory biomarkers and thus underlines the broad spectrum of action of 1,8-Cineol [94].

## 5. Conclusions

In summary, the evaluation of the current literature and data underlines the complex and holistic modes of action of 1,8-Cineol in inflammatory processes. There is tremendous evidence that the monoterpene 1,8-Cineol causes manifold anti-inflammatory and health-promoting effects in different human diseases.

Special attention must be paid to the impact of 1,8-Cineol with regard to the penetration and inhibition of bacterial biofilms, which is a major problem of antibiotic treatment in different diseases. Novel forms of 1,8-Cineol application and new insights about its systemic distribution in the human body will open new areas of its clinical use. In this context, the useful properties of 1,8-Cineol may also have supporting effects on existing treatment patterns. A better understanding of the underlying cellular and molecular regulatory mechanisms will further improve the treatment options and possible forms of application and may lead to the identification of novel therapeutic targets of this natural drug.
